# Diosmin-Loaded Nanoemulsion-Based Gel Formulation: Development, Optimization, Wound Healing and Anti-Inflammatory Studies

**DOI:** 10.3390/gels9020095

**Published:** 2023-01-22

**Authors:** Md. Khalid Anwer, Mohammed F. Aldawsari, Muzaffar Iqbal, Bjad K. Almutairy, Gamal A. Soliman, M. Ali Aboudzadeh

**Affiliations:** 1 Department of Pharmaceutics, College of Pharmacy, Prince Sattam Bin Abdulaziz University, Al-kharj 11942, Saudi Arabia; 2 Department of Pharmaceutical Chemistry, College of Pharmacy, King Saud University, Riyadh 11451, Saudi Arabia; 3 Central Laboratory, College of Pharmacy, King Saud University, Riyadh 11451, Saudi Arabia; 4 Department of Pharmacology and Toxicology, College of Pharmacy, Prince Sattam Bin Abdulaziz University, Al-kharj 11942, Saudi Arabia; 5 Department of Pharmacology, National Research Centre, Giza 12622, Egypt; 6 CNRS, Institut des Sciences Analytiques et de Physico-Chimie pour l’Environnement et les Matériaux, University Pau & Pays Adour, E2S UPPA, IPREM, UMR5254, 64000 Pau, France

**Keywords:** nanoemulsion, Box–Behnken design, gel, wound healing, anti-inflammatory activity

## Abstract

The wound-healing process is complex and prone to interruption or failure, which can result in the development of chronic wounds that never heal. This can be overcome by seeking prompt medical attention, which will reduce the likelihood of complications and speed up the healing of the cutaneous wound. It has been established that functionalized engineered biomaterials are a possible strategy for starting skin wound care. The purpose of the current study is to develop a diosmin (DSM)-loaded nanoemulsion (NE)-based gel formulation and to investigate its wound healing and anti-inflammatory activity on rats. The DSM-loaded NEs (F1-F17) were developed and optimized with the help of Box–Behnken Design Expert. The DSM-Nes were developed using lauroglycol 90 (LG90^®^) as oil, Tween-80 as surfactant and transcutol-HP (THP) as co-surfactant. The optimized Nes showed globule size (41 ± 0.07 nm), polydispersity index (PDI) (0.073 ± 0.008) and percentage of entrapment efficiency (%EE) (87 ± 0.81%). This optimized DSM-loaded NEs (F1) was further evaluated and incorporated into 1% carbopol 940 gel. F1-loaded gel was then characterized for drug content, spreadability, in vitro release, wound healing, and anti-inflammatory studies. The developed gel of DSM was found to show significantly better (*p* < 0.05) wound-healing and anti-inflammatory activity.

## 1. Introduction

Diosmin (DSM) is a flavone glycoside derived from hesperidin, a flavanone amply found in citrus fruits [1,2,3]. It was first isolated in 1925 from the Scrophularia nodosa Lamiales that belongs to Scrophulariaceae family, indigenous to the Northern hemisphere. Its therapeutic significance was first discovered in 1969, being mainly used in Europe as phlebotonics (flavonoid-antioxidant) and vascular protector for the treatment of the foot ulcer and hemorrhoids conditions [4]. To date, many biological activities have been reported for DSM such as anti-diabetic [5], anti-cancer [6], retinal protection [7], reno-protection [8], cardiovascular protection [9,10], neuroprotective [11], anti-inflammatory [12], and antioxidant properties [13]. The anti-inflammatory effects of DSM could be due to the potent reduction in the prostaglandin and thromboxane synthesis and inhibition of leukocyte activation [14].

DSM was also reported to have wound-healing activity when administered orally [15]. DSM and its metabolite diosmetin modulate the elastase and collagenase, degrading elastin and other extracellular matrix components that facilitate tissue remodeling and wound-healing activity [16]. The wound is an injury to the integrity of the biological tissue, mucous membrane, skin, and organ tissue. As per the CDC (Center for Disease Control and Prevention), wounds are classified as clean, contaminated, and dirty. A wound can be open with broken skin or closed when internal damage to tissue is covered under the intact skin. Incidence of wounds was found to be in 1–2% of the population during their lifetime in developed countries. Annually, about 13 million people suffer from chronic wound globally, and many more in the geriatric and aging populations [17,18].

During the COVID-19 pandemic, a non-healing wound is typically correlated with diabetes, hypertension, renal failures, and lethal respiratory syndromes. Pharmacoeconomic and medical cost projections for all types of wounds ranged from 28.1 to 96.8 billion USD. Wound care products have a market share of 20.6 billion USD in 2021, which is expected to grow at a 4% compound annual growth rate from 2022 to 2030 [19]. 

There is a wide variety of medication and materials available for the acute and chronic wound management. However, topical medications speed up the repair mechanism. Wound healing is a sequential process which involves hemostasis, inflammatory, proliferative and remodeling stages [20]. Effectiveness of the biological moiety of DSM could be developed by fabricating a nanocarrier that improves its permeation and sustains its therapeutic effects. DSM improves capillary permeability, microcirculation and the healing mechanism. 

Drugs can be enhanced therapeutically by being encapsulated in a way that allows for controlled release and target delivery utilizing a variety of coating materials [21,22]. In the encapsulation process, selecting the right coating material is essential because it affects target delivery and controlled release, which, in turn, affects the bioaccessibility of active ingredients. Among different choices, nanoemulsion (NE as a potential drug delivery system) allows for the controlled or sustained release of drugs for oral, topical, ocular, and percutaneous administration [23]. A recent report on NE encapsulation systems revealed their acceptance and popularity to accommodate both synthetic and herbal drug delivery to the dermal region. Nanoemulsion (NEs) is a biphasic liquid dispersion in which the globule size ranges up to 100 nm. It is a thermodynamically stable isotropic system that contains oil, water, and an emulsifier, surfactant, and co-surfactant [24,25,26]. 

DSM is practically insoluble in water, being consequently freely soluble in aprotic solvents, and shows variability in oral bioavailability and effective dermal concentration [27,28]. Percutaneous absorption of the drug is one of the major challenges due the stratum corneum of the skin acting as barrier to the entry of xenobiotic. A solubility study was performed by selecting the suitable fat-based oils; LG-90^®^, PGMC, Olive oil, sesame oil, sunflower oil, and soy were also examined for maximum solubility in the surfactant blends Tween 20, Tween 80, ethanol and Transcutol HP. 

NE carriers offer numerous advantages for controlled drug release, as well as better stability and suitability in loading it for the topical delivery of cosmetic and therapeutics. In this study, NEs were designed and optimized using design of experiment (DoE) applying the Box–Behnken design. 

Topical formulations are considered the most suitable, safe and efficient dosage form for treating skin-related diseases and conditions. Local application of medicaments also reduces the toxicity and delivers the drug in the effective concentration at the receptor site in a low dose with patient compliance. Gels are the most acceptable topical drug delivery system, with a three-dimensional network of polymers hydrated with water impregnated with the drug or drug carriers [29].

To increase bioavailability through solubilization or encapsulation and to improve the therapeutic effects, a number of DSM formulations, including gels [30], solid dispersion [31], complexation [27], nanosuspension [28], phytosomes [32], solid lipid nanoparticles [33,34], and polymer nanoparticles [35], have been investigated recently. 

To the best of our knowledge, there has not been any research reporting an NEs-based topical gel for wound healing and anti-inflammatory activities. Therefore, our investigation aimed to prepare and optimize DSM-loaded NEs with Box–Behnken design using an aqueous titration technique. Optimized NE was then loaded into the carbopol 940 gel and characterized according to its formulation parameters and release kinetics. We have evaluated the developed formulation and our findings are based on the in vivo excision wound models in rats followed by histopathological examinations.

## 2. Results and Discussion

### 2.1. Solubility Studies

**Figure 1:**
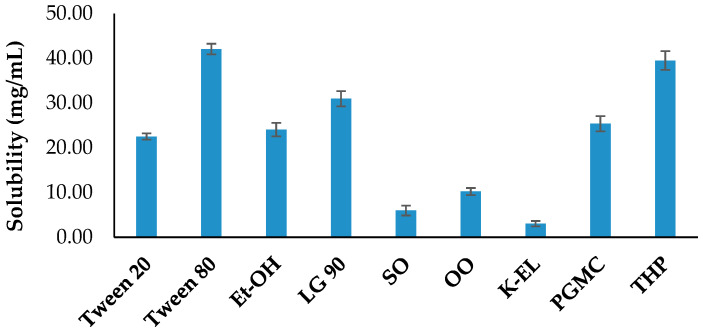
Solubility profile of DSM in different components.

Figure 1 illustrates the solubility of DSM in a few selected pure oils, surfactants, and co-surfactants. The highest solubility of DSM among different tested solvents was found in LG90 (oil), surfactant (Tween-80) and co-surfactant (THP) as 30.96 ± 1.7 mg/mL, 42.08 ± 1.2 mg/mL, and 39.52 ± 2.1 mg/mL, respectively. DSM is highly hydrophobic in nature; therefore, incorporation into NEs is useful to enhance solubility [34].

### 2.2. Experimental Design

#### 2.2.1. Effect of Independent Variables on Globule Size

(Y1) = + 40.20 − 3.99A − 1.00B − 6.75C − 0.50AB − 4.50AC − 7.50BC + 7.89A^2^ − 0.100B^2^ + 9.40C^2^

where A—Oil (%); B—Surfactant (%); and C—Co-surfactant (%)

**Figure 2:**
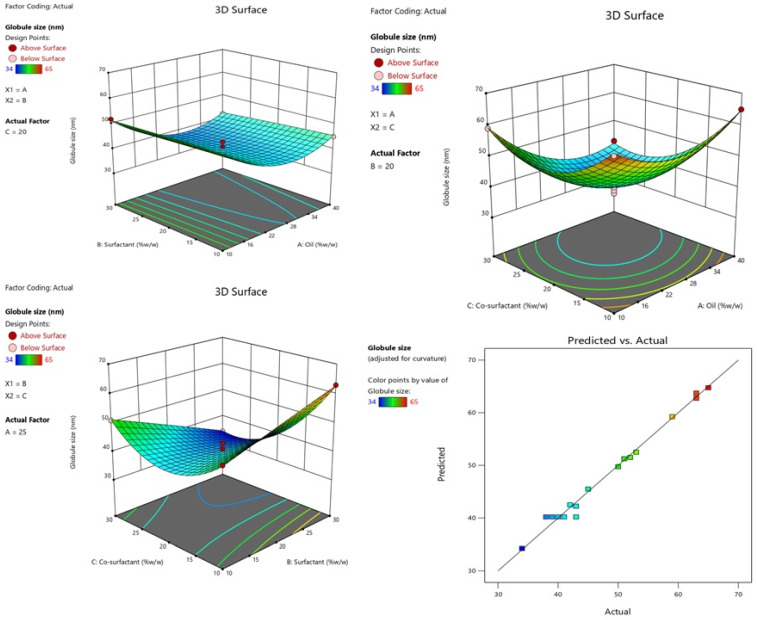
Effect of conc. of oil and surfactant and co-surfactant on globule size, and plot of and actual and predicted.

Table 1 lists the effects of independent factors on the globule sizes of various formulations. Negative coefficients of A (oil) and C (co-surfactant) significantly affect the response Y1, showing that the mean globule size was observed to decrease with decrease in the concentration of oil and co-surfactant [36] (see Figure 2). This equation showed A, C, AC, BC, A^2^ and C^2^ to be significant model terms with *p* < 0.05. The significance of the model was demonstrated by its model F-value of 66.55 (*p* < 0.05). An ANOVA evaluation of the quadratic model showed that responses Y1 and Y2 were significantly fitted (Table 2). The model performed well because the lack of fit’s F-value of 0.225 suggested that it was not significant. The quadratic model has a higher R^2^ (0.9884) than the other models. An appropriate signal was suggested by the sufficient precession larger than 4 (25.29) [37]. The predicted R^2^ and adjusted R^2^ values for response Y1 was found, with a reasonable agreement with adjusted (R^2^ = 0.9736) value, which indicates that the model has predicted the responses well [38]. When response Y1, or globule size, is given, and A, B, and C have negative coefficients, the average globule size was seen to decrease with an increase in oil, surfactant, and co-surfactant concentrations (see Figure 2). The 3D graph and plots of actual versus predicted particle size are shown in Figure 2.

#### 2.2.2. Effect of Independent Variables on PDI

**Figure 3:**
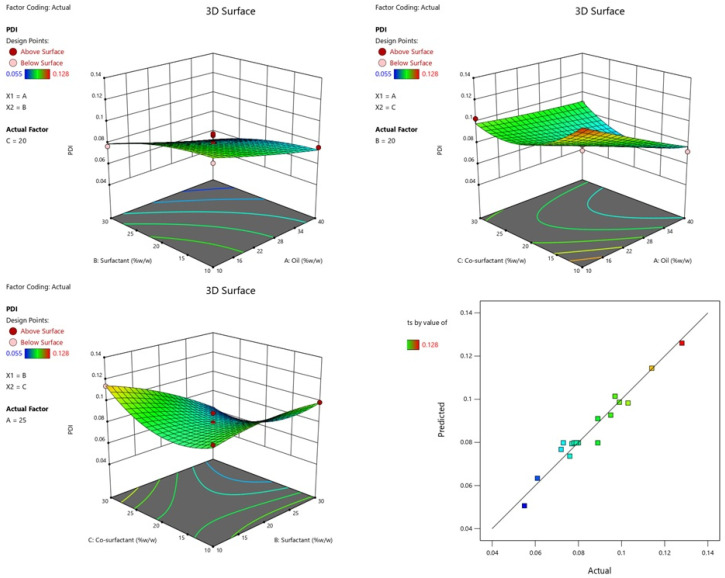
Effect of conc. of oil and surfactant and co-surfactant on PDI, and plot of actual and predicted.

Table 1 lists the influence of independent variables on the PDI of various NEs. The quadratic model was found to be the most appropriate model for the experimental design. The model is suggested to be significant by the model’s F-value of 16.42. The large F-value might happen as a result of noise only 0.06% of the time. The lack of fit was indicated by the F-value of 1.13; the lack of fit was not significant in comparison with the pure error [38]. The model was well-fitted since its R^2^ value of 0.9548 was greater than that of other (linear, second order) models. Adequate precession of 16.47 that was sufficient (>4) showed that the model was significantly fitted. ANOVA of the quadratic model was calculated and found to be significantly fitted (Table 2). The 3D graph and plots of actual versus predicted particle PDI are shown in Figure 3.

#### 2.2.3. Effect of Independent Variables on %EE

EE (Y3) = + 87.79 + 5.125A + 0.625B + 3.00C − 1.17 × 10^−14^ AB + 10.25AC + 6.25BC − 7.15A^2^ − 9.15B2 − 4.89C^2^

**Figure 4:**
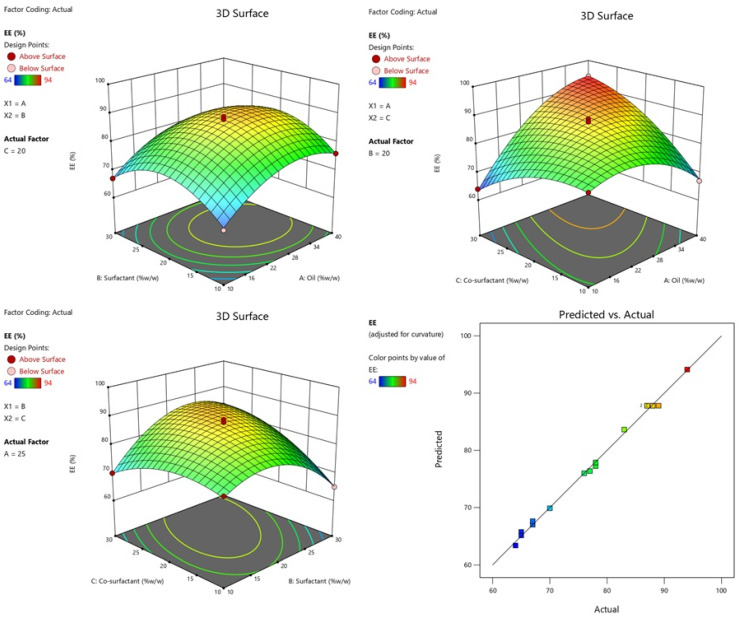
Effect of conc. of oil and surfactant and co-surfactant on %EE, and plot of actual and predicted.

The %EE of the all NEs formulae were determined and the results are listed in Table 1. The effect of independent variables were studied on %EE. The model is implied to be significant by the model’s F-value of 224.63. An F-value this large might happen due to noise only 0.01% of the time. Model terms are considered significant when the *p*-value is lower than 0.0500. In this case, the model terms A, C, AC, BC, A^2^, B^2^, and C^2^ were significant. Model terms are not significant if the value is higher than 0.1000. Model reduction may enhance your model if it has a large number of useless terms. The F-value for the lack of fit, which is 1.31, indicates that the lack of fit is not significant in comparison with pure error. A large lack of fit F-value has a 38.73% chance of being caused by noise. We want the model to fit; thus, a non-significant lack of fit is ideal (Table 2). The 3D graph and plots of actual versus predicted particle %EE are shown in Figure 4.

### 2.3. Measurement of Globule Size, PDI, %EE and Zeta Potential

**Figure 5:**
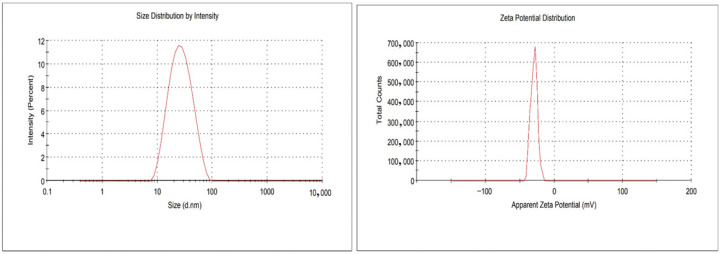
Globule size and zeta potential of optimized DSM-loaded NEs (F1).

The prepared DSM-loaded NEs were found to have a mean globule size in the range of 34–65 nm and PDI 0.055–0.128. The ideal mean globule size for topical distribution is below 200 nm, with a PDI below 0.3 [39]. This is because the globules have a large surface area that is suitable for quick pore transport and good for topical gel. The optimized DSM-loaded NEs were determined as globule size (41 nm), PDI (0.073) and ZP (−23.4 mV). This optimized DSM-loaded NEs were further incorporated into carbopol gel. The %EE of prepared DSM-loaded NEs (F1–F17) were found to be in the range of 64–94% (Table 1). Based on the results, DSM-loaded NEs (F1) were optimized with globule size (41 nm), PDI (0.073), ZP (−23.4 mV) (Figure 5) and %EE (87%).

### 2.4. Determination of Viscosity, Refractive Index (RI) and pH 

The viscosity, RI and pH of the optimized DSM-loaded NEs (F1) was measured as 77.1 ± 2.42 cP, 1.28 ± 0.03, and 4.6 ± 0.08, respectively. The ideal RI value, which demonstrates the transparency and isotropic quality of NEs formulation, was measured. It was determined that the pH was 4.6 ± 0.08, which is completely compatible with the pH of the skin and would not irritate the skin. It was found that the optimized DSM-loaded NEs (F1) have a 98.59 ± 0.03% transmittance, which is closest to 100% transmittance, indicating formulation transparency and clarity [40,41].

### 2.5. Thermodynamic Stability Studies

To check the potential of metastable or unstable NEs, thermodynamic stability was explored. After centrifugation, heating and cooling, and freeze–thaw cycles, it was found that the optimized DSM-loaded NEs (F1) were thermodynamically stable. Results of thermodynamic stability shows that the DSM-loaded NEs (F1) had a long shelf-life. The optimized DSM-loaded NEs (F1) were determined to be thermodynamically stable [42] and found to be suitable for incorporation into gel.

### 2.6. Transmission Electron Microscopy (TEM)

**Figure 6:**
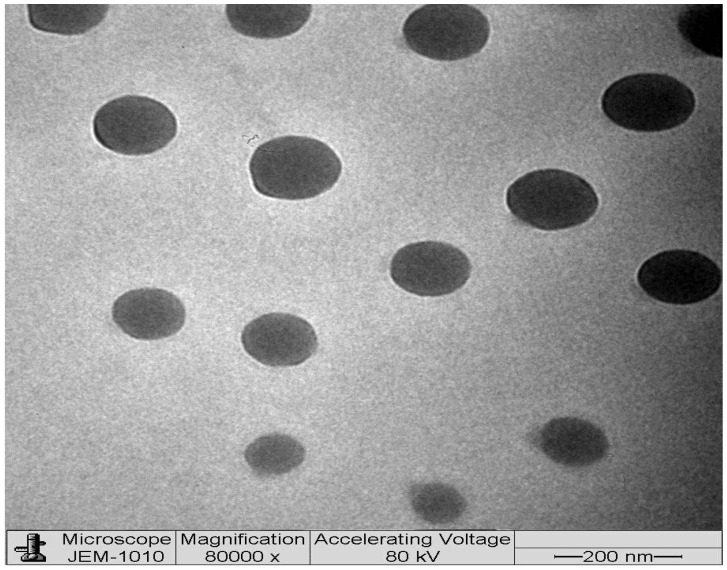
TEM images of optimized DSM-loaded NE (F1).

Using TEM, the globules of optimized DSM-loaded NEs (F1) were morphologically characterized. The TEM picture (Figure 6) shows a spherical shape and an average size of about 40 to 90 nm. The globules are evenly distributed. The majority of the particles in the TEM pictures measure less than 100 nm in size.

### 2.7. Evaluation of Gel

#### 2.7.1. pH and Drug Content

The F1-loaded gel was assessed for its pH and drug content. The gels had a pH of 6.1 ± 0.3, which is ideal for skin application. DSM content in F1-loaded gel was measured as 98 ± 4.1%. With a uniform distribution of DSM, the maximum amount of medication in the gel can be determined from the results.

#### 2.7.2. Spreadability

Excellent spreading is required to evenly cover the wound with the drug for a quicker wound-healing process [43]. The spreadability value, which was indicated in the area, is a crucial factor in how evenly the gel will spread over the skin. The area, which was determined to have a decent spreading capacity, was 6.13 ± 0.19 cm^2^.

### 2.8. In Vitro Release Studies and Kinetics 

**Figure 7:**
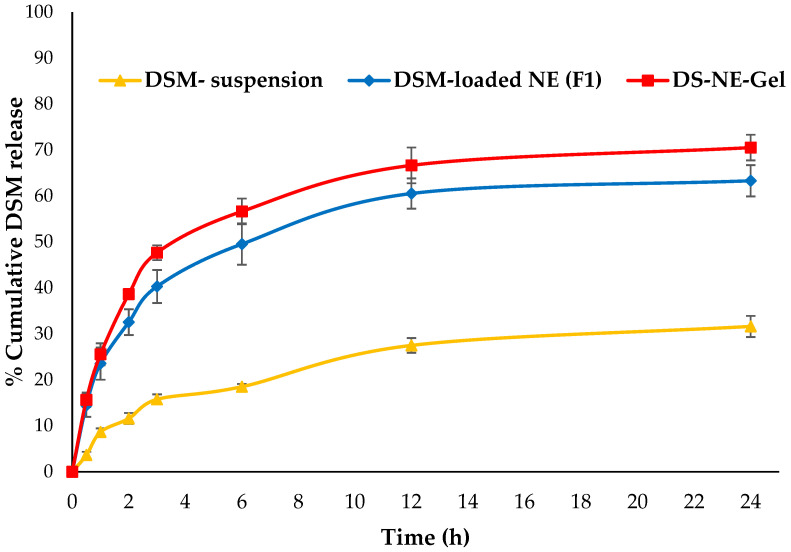
Comparative in vitro release profile.

A comparative in vitro release profile of pure DSM suspension, optimized DSM-loaded NEs (F1) and F1-loaded gel were presented in Figure 7. The carbopol gel system’s release behavior indicated a dual-release pattern; the early stages of drug release appeared to be a quick release in the first 2 h, while the later stages were a sustained release for a longer period of time (24 h). The amount of DSM released from pure DSM suspension, optimized DSM-loaded NEs (F1) and F1-loaded gel in 24 h was 28.76%, 63.32% and 70.54%, respectively. Due to its poor aqueous solubility, pure DSM demonstrated a poor release. The release was observed to be significantly increased (*p* < 0.05) following the encapsulation of DSM into NEs. Lower globule size of optimized DSM-loaded NEs (F1) is possible due to presence of Lauroglycol-90, tween-80 and THP as oil, surfactant and co-surfactant, respectively. Due to the larger surface area, smaller droplets would have a higher drug-release profile [41]. The release kinetics equations were used to study the DSM release pattern from optimized DSM-loaded NEs (F1) and F1-loaded gels. Applying the kinetic models, it was found that Korsmeyer–Peppas model could adequately describe the release of DSM from both NEs and gels. The Korsmeyer–Peppas model had the highest coefficient of correlation values among the applied release models (R^2^ = 0.9890 and 0.9793 for DSM-loaded NE (F1) and F1-gel, respectively, as shown in Table 3). The release exponents were also calculated in light of the values of slopes R^2^ obtained for the Korsmeyer–Peppas model, and the results suggested that the mechanism of drug release from DSM-loaded NE (F1) and F1-gel were of the Fickian diffusion type (Table 3) [44].

### 2.9. Acute Dermal Toxicity

The acute dermal toxicity test confirmed the safety of F1-loaded gel application. After 24 h of treatment, the shaved region showed no signs of erythema or edema. When rats were monitored for 14 days after the gel application, neither mortality nor any serious modifications in their behavior or respiratory patterns, any inability to consume food or water, or abnormal postural alignment were found, which bore close resemblance to our previous study [24]. 

### 2.10. Evaluation of Wound Healing Activity

#### 2.10.1. Excision Wound Model

**Figure 8:**
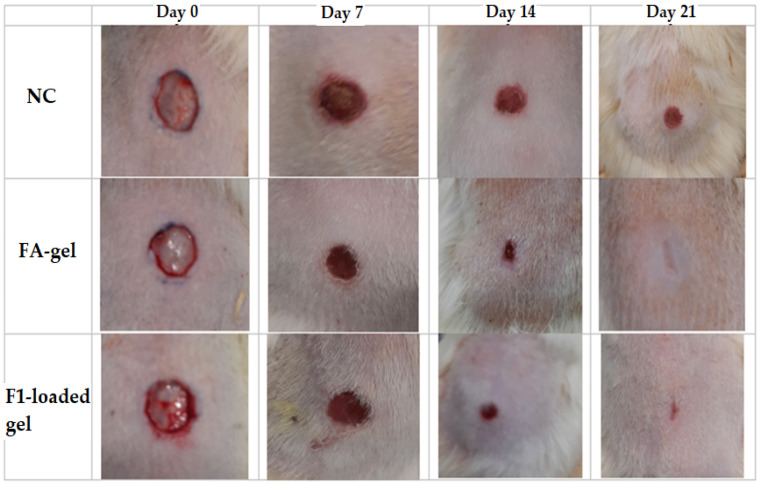
Photographs of wound healing at different time intervals in excision wound model in rats.

**Figure 9:**
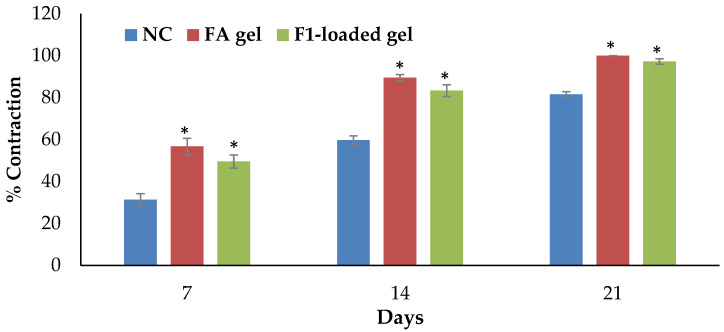
Percentage of wound contractions at various time intervals in the excision wound model in rats. Data are shown as the mean (S.E.M., n = 6 rats/group); significance is indicated by an asterisk (*) when compared to the NC group at *p* < 0.05.

In comparison with the negative control group, topical application of 2% F1-loaded gel demonstrated a substantial impact on the wound-healing process. Figure 8 depicts the progressive wound contraction induced by the use of base gel, 2% F1-loaded gel, and 2% fusidic acid gel. The 2% F1-loaded and fusidic acid gel facilitates wound contraction significantly from the 7th day to 21st day compared to NC (Figure 9).

#### 2.10.2. Microscopic Evaluation of the Wound

The photomicrograph of the NC group stained with H&E shows severe tissue damage in the form of necrosis (N) due to the absence of nuclei, degeneration (D) in the form of vacuoles, as well as occlusion of blood vessel (O) through a large spot of hyaline material. Moreover, the blood vessel walls suffer from injury, leading to hemorrhage (H). Fusidic acid gel treatment shows an improved and almost normal tissue sample but still suffering from toxic effect in the form of degeneration (D) and necrosis (N). However, F1-loaded applied on a wound shows a much improved and almost normal tissue sample. 

**Figure 10:**
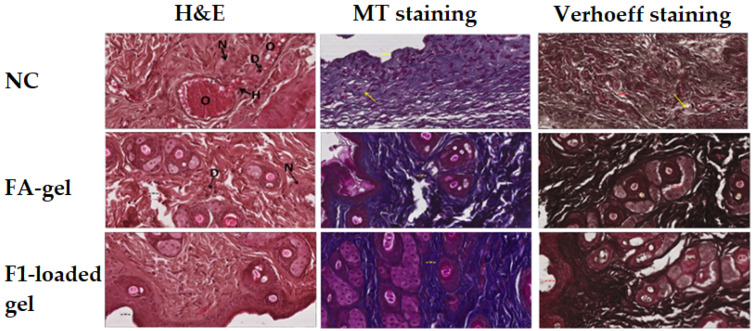
Photomicrographs of skin samples after 21 days treatment with DSM-loaded NE (F1) gel and fusidic acid gel were taken under a microscope using the Hematoxylin and Eosin (H&E), Masson Trichrom (MT), and Verhoeff stains (magnification 400×, scale bar 20 m). Abbreviations stand for necrosis (N), degeneration (D), occlusion of blood vessel (O), and hemorrhage (H) (H).

The collagen fiber loss is visible on the photomicrograph of the NC group using the MT stain as a light blue tint (example is indicated by yellow arrow). Fusidic acid-treated skin tissues exhibit increased collagen fiber condition as a dark blue color. Treatment with F1-loaded gel shows almost normal and significantly improved collagen fiber condition (Figure 10).

The photomicrograph of the NC group stained with the Verhoeff method shows the loss of elastic fibers as a lower density of black color (example is indicated by yellow arrow). In comparison with the NC group, the photomicrograph of the positive control group after treatment with fusidic acid gel and F1-loaded gel reveals an improvement in the condition of the elastic fibers (Figure 10).

### 2.11. Evaluation of Anti-Inflammatory

In Table 4, the percentages of inhibition of edema by F1-loaded gel and fusidic acid gels were shown as 42.51% and 50.69%, respectively, compared to the control. Based on these findings, it can be said that the F1-loaded gel produced an anti-inflammatory effect that was at least 1.2 times stronger than that of the fusidic gel. The F1-loaded nanogel provides highest concentration of DSM at the target side and because the drug can penetrate the skin more easily due to its nanosize, the nanogel formulation may have the strongest anti-inflammatory effects [45,46].

## 3. Conclusions

In this study, the solubility of diosmin in numerous oils, surfactants and co-surfactants was compared and the best oil, surfactant and co-surfactant blend was selected for the development of NEs. The NE was prepared through an aqueous titration method using QBD design. NE with uniform globules size, PDI and higher EE was selected for the incorporation into carbopol 940 gel. All the pharmaceuticals characterizations were considered and, after showing the optimum and fickian drug release, optimized DSM-NEs (F1) loaded gel was further tested in vivo for anti-inflammatory and wound-healing activity. The excision model wounds were examined histopathologically. On the basis of gross necropsis and histological images from the rat wound, it can be concluded that there was a faster wound-healing process observed in the test animals compared to the STD and control. Finally, in vitro and in vivo results revealed that diosmin-encapsulated nanoemulsion-loaded carbopol 940 gels would be a promising topical formulation for wound-healing and anti-inflammatory effects with minimal/acceptable acute dermal toxicity.

## 4. Materials and Methods

### 4.1. Materials

Diosmin (DSM) was purchased from Fluka Chemica (Busch, Switzerland). We received gift samples of Lauroglycol-90 (LG-90^®^), PGMC, and Transcutol^®^-HP (THP) from Gattefosse (Lyon, France). The following items were purchased from Sigma Aldrich, St. Louis, MO, USA: Kollidon-EL (K-EL), olive oil (OO), sesame oil (SO), Tween^®^-80, Tween^®^-20, ethanol, and carbopol 940.

### 4.2. Solubility Studies

To choose the best oil phase for the development of the formulation, solubility experiments were conducted. Numerous oils, including LG-90^®^, PGMC, kollidon-EL (K-EL) olive oil (OO), and sesame oil (SO) were used to test the solubility of DSM. The same experiments were performed with surfactants (tween 20, and tween 80) and co-surfactants (ethyl alcohol and Transcutol HP) [24]. Briefly, an excess amount of DSM in 2 mL of each of the selected oils, surfactants, and co-surfactants was dissolved and mixed, followed by shaking on a biological shaker (LBS-030S, Lab Tech, Jeju, Korea) for 72 h days at 37 ± 1 °C, 100 rpm. The DSM concentration in each component was measured in triplicate using HPLC at 346 nm [47].

### 4.3. Experimental Design

The development of DSM-loaded NEs was carried out utilizing a three-level Box–Behnken experimental design (BBD) with three independent factors using Design Expert software (version 13.0 Stat-Ease, Inc., Minneapolis, MN, USA). The Box–Behnken design model for response surface approach is useful because it enables: (i) evaluation of the quadratic model parameters; (ii) creation of following designs; (iii) identification of model’s lack of fit; (iv) utilization of blocks; (v) the avoidance of severe experimenting because it could lead to disappointing results. In addition, it performs fewer runs of experiments with three factors. The concentration of oil (LG^®^90; X1), concentration of surfactant (tween 80; X2), and concentration of co-surfactant (THP; X3) were the three parameters chosen for this investigation. They were grouped into three levels and assigned the codes +1, 0, and −1 for high, moderate, and low values, respectively. The oils, surfactants, and co-surfactants were selected based on solubility study. The following independent variables or components were chosen: (A) oil concentration (10–40% *w*/*w*), (B) surfactant concentration (10–30% *w*/*w*), and (C) co-surfactant concentration (10–30% *w*/*w*). The mean globule size (Y1), PDI (Y2) and entrapment efficiency (Y3) were selected as the dependent variables/responses in order to optimize the NEs. The software created a total of 17 tests, and the experiments were carried out in a random order. The independently coded variables are displayed in Table 1. Formulations were prepared and further examined for the 17 batches that were produced.

### 4.4. Selection of Optimized Formulation

The software’s point prediction, which explained the formulation’s center point, was used to select the optimum formulation. The composition of optimized NEs had oil (LG90, 25% *w*/*w*), surfactant (tween, 20% *w*/*w*) and co-surfactant (THP, 20% *w*/*w*), the predicted values of globule size (40.2 nm), PDI (0.0798) and %EE (87.8%). However, the experimental values of optimized NEs were determined to be close to the predicted values of globule size (41 ± 0.07 nm), PDI (0.073 ± 0.008) and %EE (87 ± 0.81%). This optimized formulation was further evaluated and incorporated into gel.

### 4.5. Preparation of DSM-Loaded NEs

The spontaneous emulsification method was used to develop NEs. Briefly, 10 mg of DSM were precisely weighed and added to the oil phase (LG-90^®^). The mixture was then added, together with surfactant (Tween 80) and co-surfactant (THP), and vortexed for 10 min at 250 rpm. The produced mixture was then gradually added to the aqueous phase that had been magnetically agitated. The resulting transparent NEs were further evaluated for globule size, PDI, zeta potential and %EE.

### 4.6. Measurement of Globule Size, PDI, %EE and Zeta Potential

All developed DSM-loaded NEs formulations (F1–F17) were assessed for their globule size and PDI using the dynamic light scattering (DLS) method with a Zetasizer Nano ZS device (Malvern Instruments, Worcestershire, UK). The degree of distribution and monodispersity of the globules was determined using the polydispersity index (PDI), and the PDI with <0.3 value was regarded as monodisperse [48]. Zeta potential measurements provide an indicator of the strength of repulsion and attraction between NEs globules as these detect the electric charges on the surface of globules [49]. A Malvern Zetasizer (Malvern Instruments, Worcestershire, UK) was used to measure the zeta potential of each formulation at a temperature of 25 ± 1 °C. Freshly prepared samples were diluted (1:500), put into an electrode cuvette, and their NSPs, PDI, and ZP values were assessed. The EE of DSM-loaded NEs formulations (F1–F17) were determined. The DSM-loaded NEs (0.5 mL) were appropriately diluted with methanol, centrifuged at 6000 rpm for 10 min and analyzed for content using HPLC at 346 nm [47]. The %EE of the DSM-loaded NEs formulations were estimated using the following equation:

$$\%\mathrm{EE}\;=\frac{\mathrm{Diosmin}\;\mathrm{analyzed}\;\mathrm{in}\;\mathrm{NEs}}{\mathrm{Diosmin}\;\mathrm{added}\;\mathrm{NEs}}\times 100$$

### 4.7. Determination of Refractive Index (RI) and pH 

With the help of a Brook-field viscometer, DV II ultra, RV cone and plate (Brookfield Engineering Laboratories, Inc., Middleboro, MA, USA), the viscosity of the optimized DSM-loaded NEs (F1) was assessed. The Abbe’s refractometer (Precision Testing Instruments Laboratory, Germany) was used to check the RI values of developed optimized DSM-loaded NEs (F1) at a temperature of 25 ± 2 °C. The optimized NEs had their pH and %T measured at 550 nm using a UV/Vis spectrophotometer (Jasco-UV-visible spectrophotometer, Model: V-630, Tokyo, Japan).

### 4.8. Thermodynamic Stability Studies

The developed optimized DSM-loaded NEs (F1) was examined for different thermodynamic stability studies in order to check the stability. According to the published protocol, tests on optimized DSM-loaded NEs (F1) included centrifugation, heating and cooling (three cycles between 45 °C and 25 °C), and freeze–pump–thaw cycles (three cycles between −25 °C and 25 °C) [42,50]. The visual analysis was used to evaluate the thermodynamic stability tests for phase separation, precipitation, and coalescence.

### 4.9. Transmission Electron Microscopy (TEM)

Transmission electron microscopy (TEM) was used to examine the size and shape of the optimized DSM-loaded NEs (F1) using a Jeol TEM model JEM-1101 (Tokyo, Japan). The TEM analysis provides the insight of NE morphology.

### 4.10. Preparation of DSM-Loaded NEs Gels

**Figure 11:**
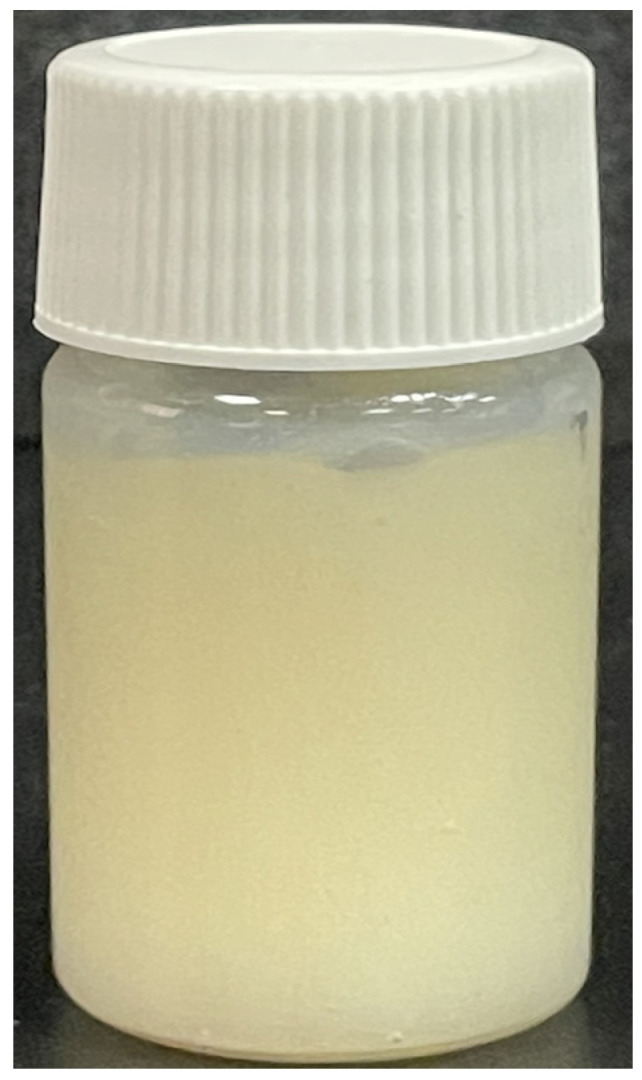
DSM-loaded NEs gel.

The carbopol 940 (1%, *w*/*v*) gelling agent was used to successfully incorporate the optimized DSM-loaded NEs (2%, *w*/*v*) into the carbopol gel. A few drops (2–3 drops) of triethanolamine (0.5%) were added to carbopol 940 dispersion to aid in the polymer’s soaking and swelling; the mixture was then kept in the fridge overnight, and, after 24 h, the optimized DSM-loaded NEs (F1) was mixed with carbopol 940 (1%, *w*/*v*) gel and methylparaben (0.1%) using magnetic stirring at a speed of 250 rpm [51]. The developed gels was further evaluated (Figure 11).

### 4.11. Evaluation of Gel

#### 4.11.1. pH and Drug Content

The F1-loaded gel, weighing about 1 g, was dissolved in milli-Q water (100 mL). The pH of the gel formulation was determined by dipping an electrode of pH meter (Adwa, Model-AD1000, Szeged, Hungary), which had been previously calibrated with standard buffers (pH 4, 7, and 9). The pH values were measured as the mean of three readings with standard deviations (Mean ± SD, n = 3). The content of DSM in the F1-loaded gel was measured by dissolving 1 g of gel in methanol (10 mL). The sample was then diluted, sonicated and analyzed for DSM content in the gel using the HPLC method.

#### 4.11.2. Spreadability

Briefly, placing the F1-loaded gel on a clean petri plate and noting its initial diameter, a second petri plate was placed on top of the first, and a weight (100 g) was placed over the second petri plate for 30 s to test the spreadability of the gel. The final dimeter was measured, and the spreadability percentage was computed using the given equation:

(1) $$\%\;Spreadability=\left(Final\;diameter-Initial\;diameter\right)\times 100$$

### 4.12. In Vitro Diffusion Studies and Release Kinetics 

To conduct in vitro release tests of the pure DSM solution, optimized DSM-loaded NEs (F1), and F1-loaded gel, Franz diffusion cells with a 10 mL capacity were used. As a diffusion membrane, the dialysis membrane (Avg. Mol wt. 14000 Dalton, Himedia, India) was soaked in phosphate buffer (PB), with a pH of 7.4, for 24 h before the experiment. The diffusion cell was filled with phosphate buffer and a dialysis membrane was attached to the cell. The assembly was kept at 37 ± 0.5 °C using a thermostatically controlled, time-programmed magnetic stirrer at 100 rpm. The samples (0.5 mL) were taken out at regular time intervals of 24 h (0, 0.5, 1, 2, 3, 6, 12 and 24 h) and replaced with an equal volume of fresh PB (pH 7.4) to maintain the sink condition. The samples were then evaluated for drug content using HPLC at 346 nm [47]. The released amount of drug was then plotted against time, and fitted to the mathematical models to obtain the release mechanism. The following model equations were used to treat release data [52]:

M_t_/M_0_ = k0.t                 Zero Order (2)

ln(M_t_/M_0_) = k_1_.t           First Order (3)

M_t_/M_0_ = k_H_.t^1/2^ s           Higuchi Model (4)

M_t_/M_0_ = K_k_. t^n^            Korsmeyer–Peppas (5)

where K is the kinetic rate constant, Mt/M_0_ is the fraction of DSM released at time t, and n is the diffusion exponent, the value of which describes the release mechanism, wherein if n = 0.5 (Fickian diffusion), 0.5 < n < 1.0 (Anomalous non-Fickian transport), and for n = 1.0 (Case-II, relaxational).

### 4.13. Experimental Animals

Healthy, Wistar albino rats (180–200 gm, 3–4 months of age) of both sexes were received from the College of Pharmacy’s Animal Care Department at the Prince Sattam Bin Abdulaziz University (PSAU), Saudi Arabia. The rats were housed in clean cages under standard laboratory conditions with a 12/12 h light/dark cycle, a temperature of 22 ± 2 °C, with an access to a pellet diet and water ad libitum. Before the experiment began, all animals were given a week to get used to the lab environment. The animal study was approved by the ethical committee of the Prince Sattam bin Abdulaziz University (SCBR-030-2022), and conducted according to the science and ethical principles for animal care, at the college of pharmacy, PSAU, Saudi Arabia.

### 4.14. Acute Dermal Toxicity

Acute dermal toxicity testing was carried out in accordance with OECD recommendation number 434. [53]. We used a total of 10 female Wistar rats (8–12 weeks). The dorsal portion of the trunk of animals with normal skin texture had about 10% of its body surface area removed 24 h before the investigation. They were divided into two groups (treatment and control). DSM-loaded gel was applied evenly to the test site, wrapped with a porous gauze bandage, and left there for a 24-h exposure period. By applying the 2% gel, a sighting study was first conducted to ascertain the starting dose. At 60 min, 24 h, 48 h, and 72 h after the initial examination, all animals were scored for the presence of erythema and edema. There were no deaths or skin rashes within 24 h, and four more rats from each group were utilized, and the same dose of gel was given. The rats were monitored daily, at least once during the first 30 min, frequently during the first 24 h, and with special attention given during the first 4 h, unless they were found dead, or any adverse dermal reaction appeared right after dosing.

### 4.15. Evaluation of Wound Healing Activity

#### 4.15.1. Excision Wound Model

The excision model was conducted on three groups of six rats each. Animals in group I (NC) were treated as negative control and given topically applied 2% base gel. Groups II (the treated group) and III (the positive control group) received, respectively, 2% *w*/*w* DSM-loaded NE (F1) gel and fusidic acid (FA) gel. 

The rats were anesthetized with chloral hydrate (400 mg/kg, IP). The animals’ dorsal furs were machine-shaved before the wound area was prepared. Then, their dorsothoracic region’s fur was cut off. Permanent marker was used to create a 200 mm^2^ circular mark, which was then completely excised to create a wound using sterile forceps and scissors. This was considered day 0. For the examination of wound-healing activity, the rats were given base, DSM-loaded (F1) gel, and fusidic acid gel starting on day one. The test groups’ wounds were treated with all of the preparations daily until they had fully healed. Every seven days, the wound area was measured using a transparent sheet and permanent pen. On graph paper with a 1 mm^2^ scale, the transparent sheet was laid down and sketched out. The wound-healing activities were assessed according to the period of epithelialization and the percentage of wound contraction [54,55]. The percentage of wound contraction was calculated as follows:

(6) $$\%\;\mathrm{Wound}\;\mathrm{Contraction}=\frac{\mathrm{Wound}\;\mathrm{Area}\;\mathrm{on}\;\mathrm{day}\;0-\mathrm{Wound}\;\mathrm{Area}\;\mathrm{on}\;\mathrm{day}\;n}{\mathrm{Wound}\;\mathrm{Area}\;\mathrm{on}\;\mathrm{day}\;0}\times 100$$

where n is the number of measurement-taking days.

#### 4.15.2. Microscopic Evaluation of the Wound

Skin tissue samples were collected from all the groups into suitable bottles. Immediately after collection, these samples were immersed in a solution of 10% formalin for fixation. Then, tissue processing was accomplished using an automatic machine for tissue processing (ASP300s, Leica Biosystems, Deer Park, IL, USA). Then, using a rotary microtome, blocks of paraffin wax were used to implant tissue samples and cut these into thin sections of 5 µm thickness (SHUR/Cut 4500, TBS, Durham, NC, USA) [50]. Three sections of each tissue block were taken for staining. One section was stained using the common staining procedure of hematoxylin and eosin (H&E). The second section was stained using the Masson tricrome (MT) special stain method for connective tissue fibers; and the third specimen was stained using the Verhoeff special stain method [50,51]. After proper staining, a histopathologist evaluated photomicrographs for pathologies and examined wound changes under a light microscope.

### 4.16. In Vivo Anti-Inflammatory Activity 

The DSM-loaded NE (F1) gel’s anti-inflammatory efficacy was tested using a rat model of carrageenan-induced hind paw edema [56,57,58]. Rats were chosen for each group so that the groups’ average body weights were as similar as possible. The animals that had been fasted for the previous night (n = 18) were grouped into three groups of six rats each to assess the anti-inflammatory activity. The plantar surface of the left hind paw was topically applied to the rats in group 1 (NC) by gently rubbing the 1% base gel 50 times with the index finger. F1-loaded gels and fusidic acid gel were given to Group 2 (the treated group) and Group 3 (the positive control group), respectively, topically on the plantar surface of the left hind paw with the same mode of application as the base gel. After 3 h since dosing, 0.1 mL of a 1% carrageenan solution (in normal saline) was injected subplantarly into the treated paw. Using a plethysmometer, the paw edema was measured immediately (0 h) and again 3 h after the carrageenan injection. The following equation was used to determine the percentage of the paw’s swelling:

(7) $$\%\;\mathrm{Swelling}=\frac{V-\mathrm{Vi}}{\mathrm{Vi}}\times 100$$

(8) $$\%\;\mathrm{Paw}\;\mathrm{Edema}\;\mathrm{Inhibition}=\frac{\%\;\mathrm{Swelling}\;\mathrm{of}\;\mathrm{control}\;\mathrm{group}-\%\;\mathrm{Swelling}\;\mathrm{of}\;\mathrm{treated}\;\mathrm{group}}{\%\;\mathrm{Swelling}\;\mathrm{of}\;\mathrm{control}\;\mathrm{group}}\times 100$$

where Vi is the initial paw volume and V is the paw volume 3 h after the carrageenan injection. The average amount of paw edema in the drug-treated group was compared to that in the control group, and the percentage inhibition of edema caused by the treatment was computed using the formula below.

### 4.17. Statistical Analysis

The mean and standard error of the mean (SEM) were used to express all the results. One-way analysis of variance (ANOVA), followed by the Bonferroni test, was used to statistically analyze the results. The results were deemed statistically significant at a *p*-value of <0.05 at 95% confidence interval (CI). All data processing was carried out using the GraphPad program (Graph Pad, San Diego, CA, USA).

## Figures and Tables

**Table 1:** Observed responses of different NEs formulae based on independent variables.

Runs	A:Oil %*w*/*w*	B:Surfactant %*w*/*w*	C:Co-Surfactant %*w*/*w*	Globule Size nm (Y1)	PDI (Y2)	EE % (Y3)
F1	25	20	20	41 ± 0.07	0.073 ± 0.008	87 ± 0.81
F2	25	10	10	50 ± 1.22	0.095 ± 0.007	77 ± 0.79
F3	40	20	30	43 ± 0.91	0.089 ± 0.001	94 ± 1.61
F4	10	30	20	52 ± 0.99	0.077 ± 0.004	67 ± 0.93
F5	25	30	10	63 ± 1.42	0.099 ± 0.001	65 ± 1.09
F6	10	10	20	53 ± 0.82	0.097 ± 0.005	65 ± 0.14
F7	25	10	30	51 ± 0.07	0.114 ± 0.011	70 ± 1.05
F8	25	20	20	38 ± 0.69	0.08 ± 0.003	88 ± 2.06
F9	25	20	20	43 ± 1.05	0.079 ± 0.001	87 ± 1.53
F10	40	30	20	42 ± 0.78	0.055 ± 0.002	78 ± 0.72
F11	25	20	20	40 ± 0.92	0.078 ± 0.001	88 ± 0.76
F12	40	20	10	65 ± 1.21	0.072 ± 0.004	67 ± 0.67
F13	10	20	30	59 ± 1.10	0.103 ± 0.009	64 ± 1.24
F14	10	20	10	63 ± 0.55	0.128 ± 0.022	78 ± 2.54
F15	40	10	20	45 ± 0.74	0.076 ± 0.001	76 ± 0.83
F16	25	20	20	39 ± 0.06	0.089 ± 0.012	89 ± 1.44
F17	25	30	30	34 ± 0.43	0.061 ± 0.002	83 ± 2.41

**Table 2:** ANOVA results for responses: globule size, PDI and %EE.

Source	Sum of Square	DF	Mean Square	F-Value	*p*-Value	
Globule size						
Model	1480.23	9	164.47	66.55	<0.0001	Significant
A—Oil	128.00	1	128.00	51.79	0.0002	Significant
B—Surfactant	8.00	1	8.00	3.24	0.1150	Not significant
C—Co-surfactant	364.50	1	364.50	147.49	<0.0001	Significant
AB	1.0000	1	1.0000	0.4046	0.5449	Not significant
AC	81.00	1	81.00	32.77	0.0007	Significant
BC	225.00	1	225.00	91.04	<0.0001	Significant
A^2^	262.78	1	262.78	106.33	<0.0001	Significant
B^2^	0.0421	1	0.0421	0.0170	0.8998	Not significant
C^2^	372.04	1	372.04	150.54	<0.0001	Significant
Residual	17.30	7	2.47			
Lack of Fit	2.50	3	0.8333	0.2252	0.8746	Not significant
Pure Error	14.80	4	3.70			
Cor Total	1497.53	16				
PDI						
Model	0.0053	9	0.0006	16.42	0.0006	Significant
A—Oil	0.0016	1	0.0016	44.86	0.0003	Significant
B—Surfactant	0.0010	1	0.0010	28.46	0.0011	Significant
C—Co-surfactant	0.0001	1	0.0001	2.56	0.1535	Not significant
AB	2.500 × 10^−7^	1	2.500 × 10^−7^	0.0070	0.9355	Not significant
AC	0.0004	1	0.0004	12.40	0.0097	Significant
BC	0.0008	1	0.0008	22.83	0.0020	Significant
A^2^	5.095 × 10^−6^	1	5.095 × 10^−6^	0.1432	0.7163	Not significant
B^2^	0.0001	1	0.0001	2.56	0.1537	Not significant
C^2^	0.0012	1	0.0012	34.61	0.0006	Significant
Residual	0.0002	7	0.0000			
Lack of Fit	0.0001	3	0.0000	1.13	0.4370	Not significant
Pure Error	0.0001	4	0.0000			
Cor Total	0.0055	16				
%EE						
Model	1602.92	9	178.10	224.63	<0.0001	Significant
A—Oil	210.13	1	210.13	265.02	<0.0001	Significant
B—Surfactant	3.12	1	3.12	3.94	0.0875	Not significant
C—Co-surfactant	72.00	1	72.00	90.81	<0.0001	Significant
AB	0.0000	1	0.0000	0.0000	1.0000	Not significant
AC	420.25	1	420.25	530.05	<0.0001	Significant
BC	156.25	1	156.25	197.07	<0.0001	Significant
A^2^	215.25	1	215.25	271.49	<0.0001	Significant
B^2^	352.52	1	352.52	444.61	<0.0001	Significant
C^2^	101.09	1	101.09	127.51	<0.0001	Significant
Residual	5.55	7	0.7929			
Lack of Fit	2.75	3	0.9167	1.31	0.3873	Not significant
Pure Error	2.80	4	0.7000			
Cor Total	1608.47	16				

**Table 3:** Release kinetics mechanism of DSM-loaded NE (F1) and F1-gel.

Code	Zero Order	First Order	Higuchi Model	Korsmeyer Peppas	Release Exponents
	R^2^	R^2^	R^2^	R^2^	n
F1	0.6013	0.8148	0.9825	0.9890	0.442
F1-gel	0.6119	0.8625	0.9751	0.9793	0.452

**Table 4:** Comparative anti-inflammatory activity on carrageenan-induced paw edema.

Groups	Percent Swelling	Percent Inhibition
NC	38.47 ± 1.51	-
FA gel	22.11 ± 1.90 ***	42.51
F1-loaded gel	18.97 ± 2.20 ***	50.69

Values are expressed as mean ± S.E.M., n = 6 rats/group, symbol *** indicates significance compared to NC group at *p* < 0.05.

## Data Availability

The data presented in this study are available on request from the corresponding author.
